# Effect of soft skill training on communication abilities among nursing students

**DOI:** 10.6026/973206300214404

**Published:** 2025-12-15

**Authors:** Monika Gaman, Jyoti Sarin, Simarjeet Kaur, Dhanesh Garg

**Affiliations:** 1 Department of Medical Surgical Nursing, Maharishi Markandeshwar College of Nursing, Maharishi Markandeshwar (Deemed to be University), Mullana, Haryana, India; 2 Department of Child Health Nursing, Maharishi Markandeshwar Maharishi Markandeshwar College of Nursing, Maharishi Markandeshwar (Deemed to be) University, Mullana, Haryana, India; 3 Department of Obstetrics and Gynaecological Nursing, Maharishi Markandeshwar College of Nursing, Maharishi Markandeshwar (Deemed to be University), Mullana, Haryana, India; 4 Department of Statistics, Maharishi Markandeshwar College of Nursing, Maharishi Markandeshwar (Deemed to be University), Mullana, Haryana, India

**Keywords:** Effectiveness, soft skill training, telephone etiquettes, professional etiquettes, nursing students

## Abstract

Insufficient soft skill training, particularly in communication abilities such as telephone and professional etiquette, among nursing students is critical in healthcare management. Therefore, it is of interest to explore the effectiveness of soft skill training in enhancing communication abilities among nursing students, focusing on telephone and professional etiquette. A quasi-experimental design was designed with 112 second-year B.Sc. nursing students, divided into experimental and comparison groups. The experimental group received targeted soft skill training, while the comparison group underwent regular clinical training. Post-test results showed significant improvements in telephone and professional etiquette scores for the experimental group. Thus, the importance of integrating soft skills into nursing education to improve patient care and professional interactions is essential.

## Background:

Skills form the cornerstone of career success, enabling individuals to perform tasks effectively, overcome challenges, seize opportunities and leave a lasting impact on various aspects of life [1]. These skills are broadly categorized into hard and soft skills. Hard skills are task-specific and necessary for executing particular job functions, while soft skills are interpersonal attributes that enable individuals to communicate, collaborate and build positive relationships with others [2]. According to research by Harvard and Stanford Universities, hard skills contribute to only 15% of career success, while 85% is attributed to soft skills [3]. Soft skills encompass a wide range of abilities such as communication, teamwork, negotiation, conflict resolution and problem-solving. These skills are vital for personal and professional success, as they enable individuals to interact effectively in various situations [4]. Numerous studies have identified over 87 soft skills that are essential for individuals to thrive in today's world. Moreover, the development of good etiquette, including telephone and professional manners, is critical for fostering trust, creating a positive image and building strong interpersonal relationships [5]. In nursing, the importance of soft skills is particularly significant, as the profession requires continuous collaboration with multidisciplinary teams, including fellow nurses, physicians and other healthcare professionals [6]. Effective communication, a crucial subset of soft skills, plays a pivotal role in establishing and maintaining healthy relationships with patients and colleagues [7]. Over the years, the perception of soft skills has evolved, with soft skills now recognized as vital in the nursing field. Traditionally, hard skills were prioritized, but the modern view acknowledges the essential role of soft skills in delivering quality healthcare [8]. Despite the growing recognition of the importance of soft skills in nursing, the quality of nursing care has been declining in some areas, with reduced time for patient interaction and communication. This issue has been linked to the decrease in patient satisfaction, as soft skills greatly influence how patients perceive their healthcare experience [9]. As a result, nursing curricula have been revised to include mandatory and elective modules focused on developing soft skills, ensuring that nursing students are well-prepared to excel not only in clinical proficiency but also in building meaningful connections with patients and colleagues [10]. Enhancing soft skills in nursing students is a continuous process that extends beyond formal training, involving role modeling and mentorship [11]. By integrating soft skill development into the nursing curriculum and providing ongoing training, nurse leaders can ensure that future nurses are equipped with the tools to provide high-quality care [12]. Therefore, it is of interest to assess the effectiveness of soft skill training in improving telephone and professional etiquettes among nursing students, ultimately contributing to their overall professional growth and better patient care.

## Methodology:

The current study adopted a quasi- experimental design (non- equivalent pre- test post- test control group design) and included 112 B.Sc. Nursing 2nd year students of M.M. College of Nursing and M.M. Institute of Nursing, Mullana, Ambala during the month of October, 2023. Power analysis was done by using Cohen's d formula, d= (µ1-µ2)/σ = 0.73, the estimated effect size was 0.73 at the power of 0.90 suggesting a recommended sample size of 43 for each group. Considering potential attrition, the researcher aimed for a sample size of 120 nursing students. However, after exclusions- 3 from the experimental group and 5 from the comparison group, the final sample comprised 112 participants, with 57 in the experimental group and 55 in the comparison group. Ethical approval for the study (IEC-2382) and (CTRI/2023/09//057349) was obtained from the Institutional Ethical Committee (IEC) of Maharishi Markandeshwar (Deemed to be University), Mullana, Ambala, Haryana, as well as from the Clinical Trials Registry-India (CTRI). The formal administrative approval was obtained from the concerned authorities of M.M. College of Nursing and M.M. Institute of Nursing, MMDU, Mullana (Ambala). The study included B.Sc. Nursing 2nd year (3rd semester) students, with those absent during data collection being excluded from participation. The sample was selected using purposive sampling technique, while the random allocation of nursing students into experimental and comparison groups was achieved through a lottery method. Data collection was done using two tools. Firstly, student profiles were gathered through a Google Form (E-filling), encompassing details such as age, gender, marital status, educational background, residency area, family status, prior knowledge of soft skills and participation in any soft skills webinars/workshops. Secondly, telephone etiquettes were assessed across three distinct case scenarios- nurse-doctors, nurse-nurse and nurse-teacher telephonic conversations- utilizing a Telephone Etiquette Checklist consisting of ten (10) statements. Additionally, professional etiquettes were evaluated through a Professional Etiquette Assessment Tool featuring eight (08) video-based stations depicting both professional and unprofessional behaviors. After establishing rapport with nursing students, informed consent was obtained from all study participants, who were assured of the confidentiality of their responses. The final data collection took place in October 2023 among B.Sc. Nursing 2nd year students. From day 1 to 3, the pre-test was conducted for the comparison group by administering the tools (Student's profile, Telephone Etiquette Checklist and Professional Etiquette Assessment Tool). Similarly, on days 18 to 20, the post-test for the comparison group was administered using the same tools. For the experimental group, from day 1 to 3, the pre-test was conducted using the same tools. Then, on day 4, soft skill training was administered to the experimental group through power point presentation, videos and case scenarios related to telephone and professional etiquettes. Finally, from days 18 to 20, the post-test for the experimental group was conducted using the aforementioned tools. Data was analysed according to the objectives, hypotheses of the study and opinion of the expert, it was planned to organize, tabulate and interpret the data by using both descriptive and inferential statistics by using SPSS 20 software after checking normality of the data by Kolmogorov- Smirnov Test. Data was normally distributed and parametric test were applied for data analysis.

## Results:

A total of 112 students completed the study. The computed chi-square values for the student's profile in experimental and comparison group for age, gender, marital status, board of education, family status, previous knowledge on soft skills and attended any webinar/ workshop/ training program on soft skills were found to be statistically non-significant except area of residence (p=0.01) which was found to be significant at 0.05 level of significance. The majority (91.22%) and (87.27%) of nursing students belong to age group of 18-20 years in experimental and comparison group respectively. More than three- fourth (78.94%) and (65.45%) of nursing students were females in experimental and comparison group respectively. Majority (66.66%) and (81.81%) of nursing students had not attend any webinar/ workshop/ training program on soft skills in experimental and comparison group respectively (Table 1 - see PDF). The independent t-test showing comparison of experimental and comparison group in terms of post-test telephone etiquettes and professional etiquettes scores among nursing students. The calculated t value was found to be (2.69 and 3.71) with p value (0.01 and 0.001) for telephone etiquettes and professional etiquettes scores respectively which was found to be statistically significant at 0.05 level of significance (Table 2 - see PDF). The paired t-test showing comparison of experimental and comparison group in terms of pre- test and post-test telephone etiquettes and professional etiquettes scores among nursing students. The calculated t value was found to be (3.45, 6.77) with p value (0.001) for telephone etiquettes and professional etiquettes scores of nursing students in experimental group respectively which was found to be statistically significant at 0.05 level of significance (Table 2 - see PDF). In experimental group, the findings showed that there was moderate correlation (r=0.48) between post-test telephone etiquettes and post-test professional etiquettes scores which was found to be highly significant (p=0.001) at 0.05 level of significance. In comparison group, there was moderate correlation (r=0.33) between post-test telephone etiquettes and post-test professional etiquettes scores which was found to be significant (p=0.01) at 0.05 level of significance (Table 3 - see PDF).

## Discussion:

The findings of the study were discussed with reference to the results obtained in other related research studies. The purpose of this present study was to assess the effectiveness of soft skill training in terms of telephone etiquettes and professional etiquettes of nursing students in selected nursing college of Haryana. In the present study, the majority (91.22%) and (87.27%) of nursing students belong to age group of 18-20 years in experimental and comparison group respectively. More than three- fourth (78.94%) and (65.45%) were females in experimental and comparison group respectively. Less than two- third (61.40%) were from urban area in experimental group whereas in comparison group more than two- third (67.27%) of nursing students were from rural area. Majority (77.19%) and (67.27%) of nursing students had some previous knowledge on soft skills in experimental and comparison group respectively. Moreover, more than half (58.2%) were residing in the urban areas [14]. The study findings were consistent with the study results carried out by Yousef *et al.* (2020) [12], where more than half (53.3%) of the participants had less than 24 years of age, majority (61.1 %) of them were females and majority (60%) of the participants were residing in the urban areas. The majority (70%) of the participants had no previous training on soft skills [15]. In the present study, the mean post-test telephone etiquettes scores (22.44±3.61) was higher in experimental group than the mean post-test telephone etiquettes scores (20.51±3.96) in comparison group. These findings were consistent with the findings of study conducted by Hussein *et al.* (2021) [13], which revealed that the head nurses' soft skills had improved before and after the training program in terms of communication with staff (94%) and communication with patients (96%) respectively. In the present study, the mean post-test professional etiquettes scores (18.02±6.39) was higher in experimental group than the mean post-test professional etiquettes scores (14.11±4.58) in comparison group. These findings were consistent with the findings of study conducted by Duba *et al.* [14], where majority of the respondents gave top priority for goal setting & work ethics and effective communication skills at workplace respectively which implies that there was a need of various soft skills training to appropriately equip students for their career success. In the present study, the findings revealed that there was significant correlation between pre-test and post-test telephone etiquettes and professional etiquettes scores among nursing students which was found to be statistically significant at 0.05 level of significance in experimental and comparison group. Moreover, these findings are consistent with the findings of study carried out by Aladwan *et al.* (2023) [15] and Mercan *et al.* (2023) [16], which stated that there was a positive correlation between the majority of soft skills dimensions and students' achievement grade score in clinical areas.

We faced a limitation in assessing professional etiquettes among nursing students in a real setting, as not all the attributes of the professional etiquette assessment tool were covered during the tool try-out. In terms of recommendations, the study can be replicated on a larger scale to investigate whether the significant findings can be sustained across a broader group. Additionally, a similar study could assess the effectiveness of soft skill training in areas such as presentation skills; telephone etiquette, professional and personal etiquettes among nursing personnel. A cross-sectional study could be undertaken to assess soft skills among nursing students throughout their course of study. Furthermore, a developmental study could focus on developing a standardized tool to assess soft skills among nursing students. An exploratory study may also be useful in identifying barriers to the implementation of soft skill training programs. Finally, a descriptive study could be conducted to understand the perception of nursing personnel regarding the importance of attending continuing education sessions such as workshops, conferences and training programs related to soft skills.

## Conclusion:

Soft skill training effectively improved telephone and professional etiquette among nursing students. These improvements highlight the need to integrate soft skills into nursing education. Such training is essential for enhancing communication and delivering high-quality patient care.
